# Supplementary material for: Response inhibition in premotor cortex corresponds to a complex reshuffle of the mesoscopic information network
Source: Netw Neurosci. 2024 Jul 1;8(2):597–622. doi: 10.1162/netn_a_00365 (PMC11168728; doi:10.1162/netn_a_00365)
Supplement: Supplementary file 1 [file netn-8-2-597-s001.pdf]

Supplementary Material for
Response inhibition in premotor cortex corresponds to a complex reshuffle of the mesoscopic information network

Giampiero Bardella[1*], Valentina Giuffrida[2], Franco Giarrocco[2], Emiliano Brunamonti[1], Pierpaolo Pani[1], and Stefano Ferraina[1]

[1]Department of Physiology and Pharmacology, Sapienza University of Rome, Piazzale Aldo Moro 5, 00185, Rome, Italy
[2]PhD Program in Behavioral Neuroscience, Sapienza University of Rome, Piazzale Aldo Moro 5, 00185, Rome, Italy
[*]corresponding author(s): Giampiero Bardella (giampiero.bardella@uniroma1.it)

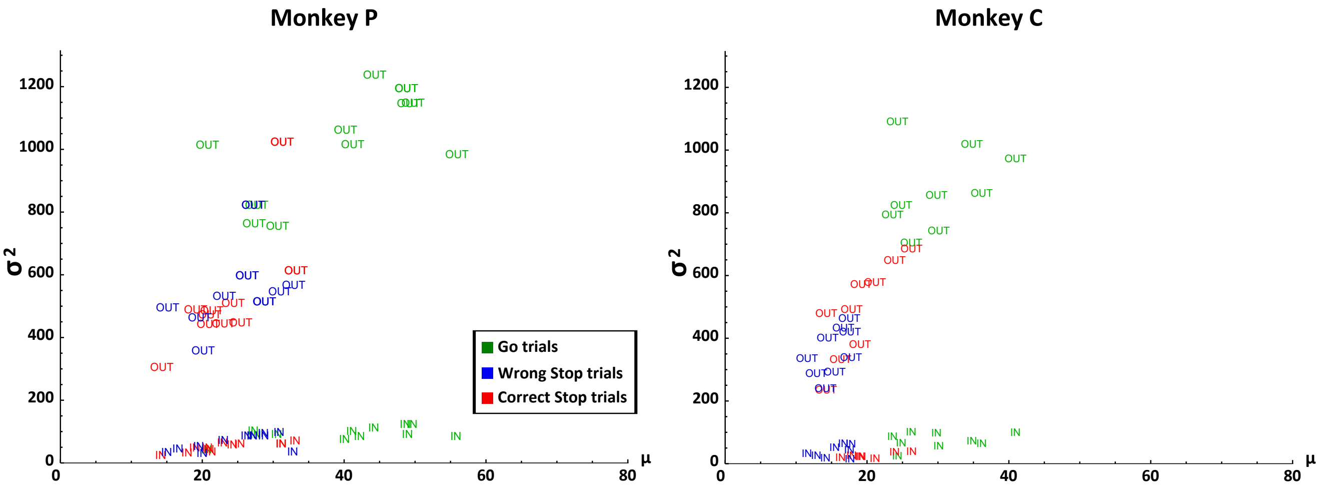

Figure S1. VD_{in} (IN markers) and VD_{out} (OUT markers) distribution parameters (x axis: mean, μ; y axis: variance, σ^2) of the PMd empirical information network across behavioral conditions for each recording session for both monkeys. As evident, only the VD_{out} distributions showed a great excursion of the variance with respect to the mean, i.e. show the presence of a fat-tail. Indeed, for the VD_{out} distributions σ^2 is at least one order of magnitude greater than μ (Kruskal-Wallis, $p < 0.01$). This documents the presence of a fat tail and hence of high out degree nodes. On the contrary, the VD_{in} distributions showed comparable values.

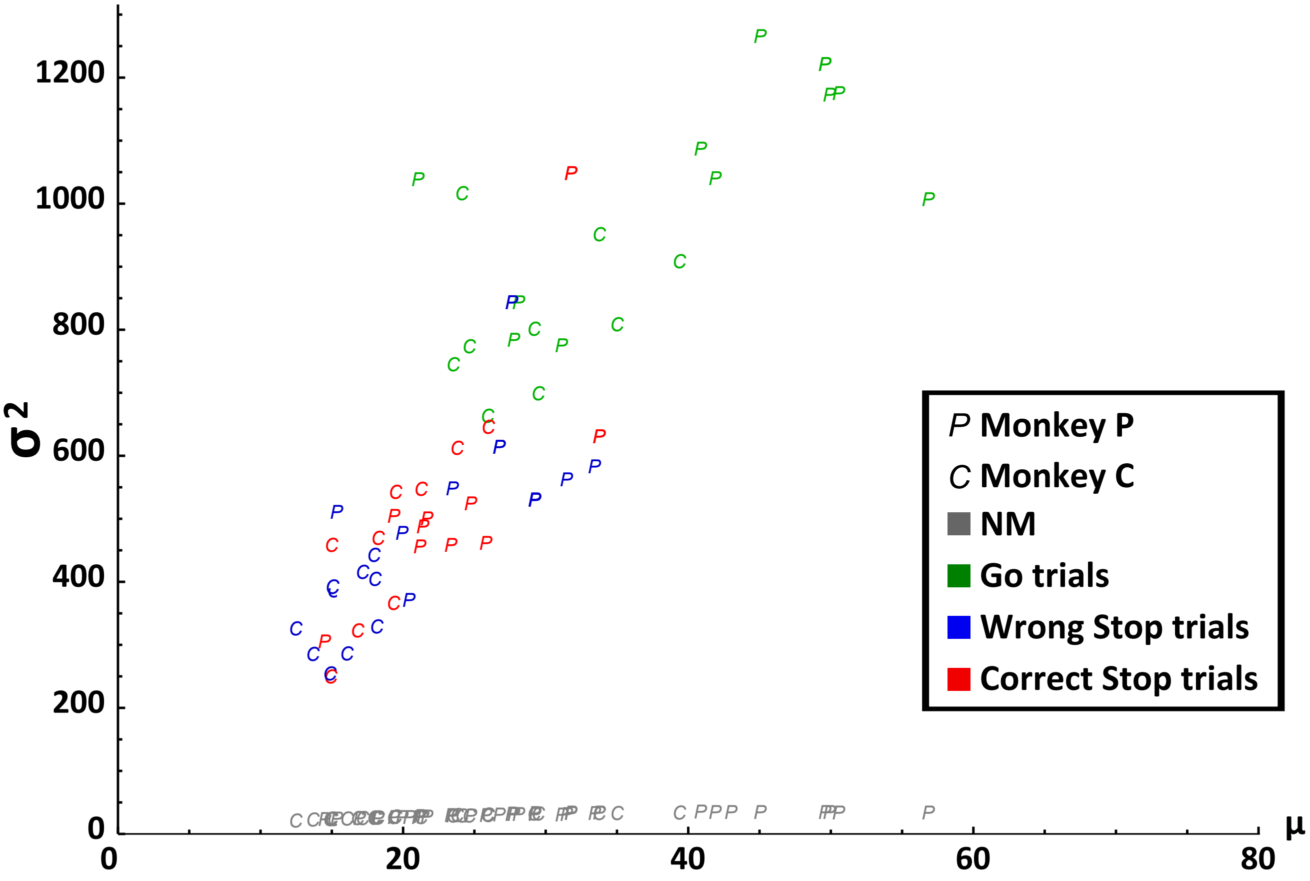

Figure S2. Comparison between the VD_{out} distribution parameters (x axis: mean, μ; y axis: variance, σ^2) of the PMd empirical information network across behavioral conditions for each recording session for both monkeys and the ensemble-average obtained from the null model (see text for details). The empirical distributions have a variance that spans values at least one order of magnitude greater than that of the networks derived from the MUA distribution, despite having comparable μ values; Kruskal-Wallis, $p < 0.01$, Bonferroni corrected for multiple comparisons. (**NM**: Null model).

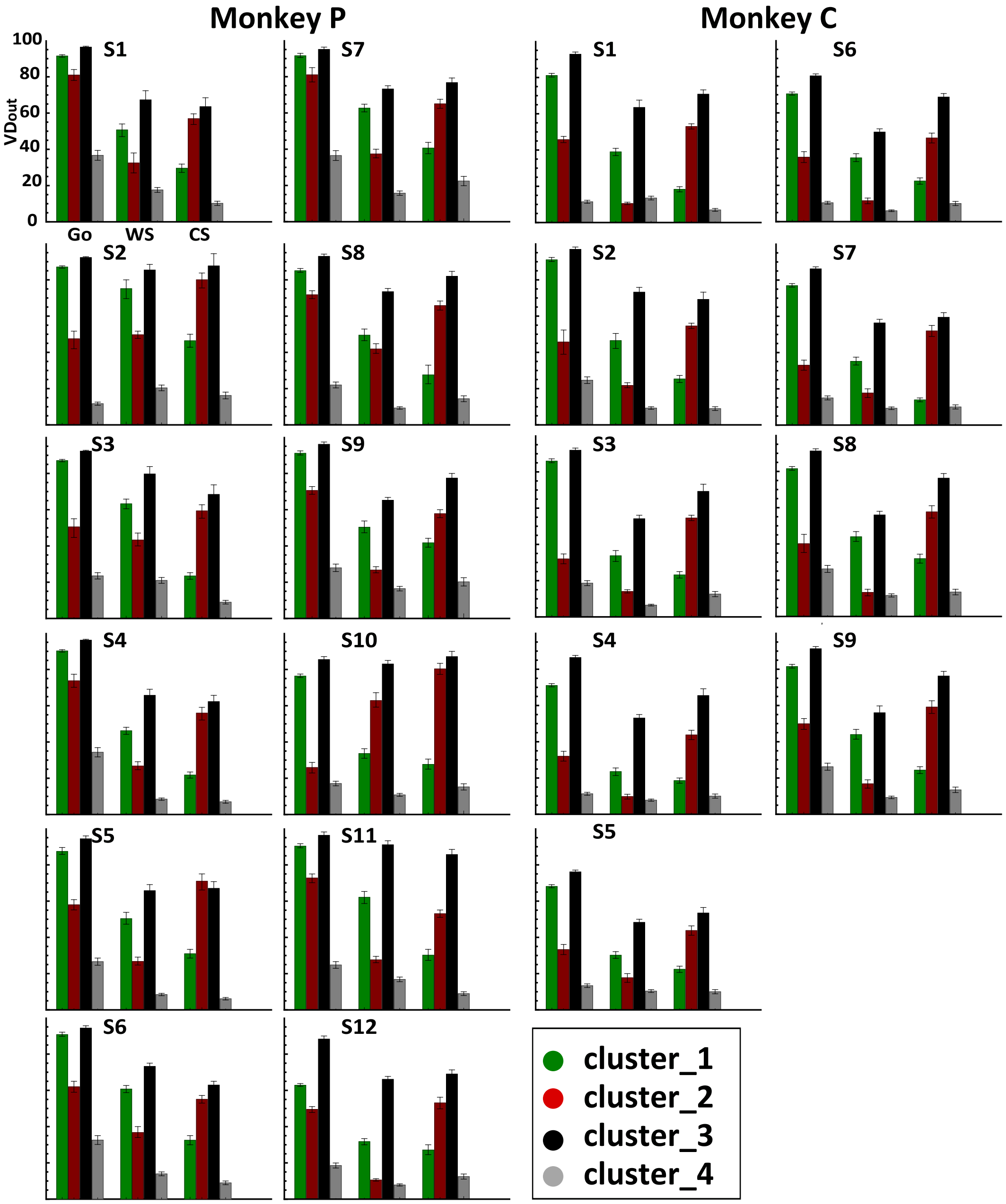

Figure S3. Topology of information transmission at the single-session level. VD_{out} values compared between clusters and across behavioural conditions. Colours reflect the neuronal clusters (see legend). Error bar is the SEM of the nodes forming the cluster based on Kruskal-Wallis, all $p < 0.01$ Bonferroni corrected for multiple comparisons. Table S5 reports the average over sessions for each cluster. (**Go**: Go trials; **WS**: wrong Stop trials; **CS**: correct Stop trials)

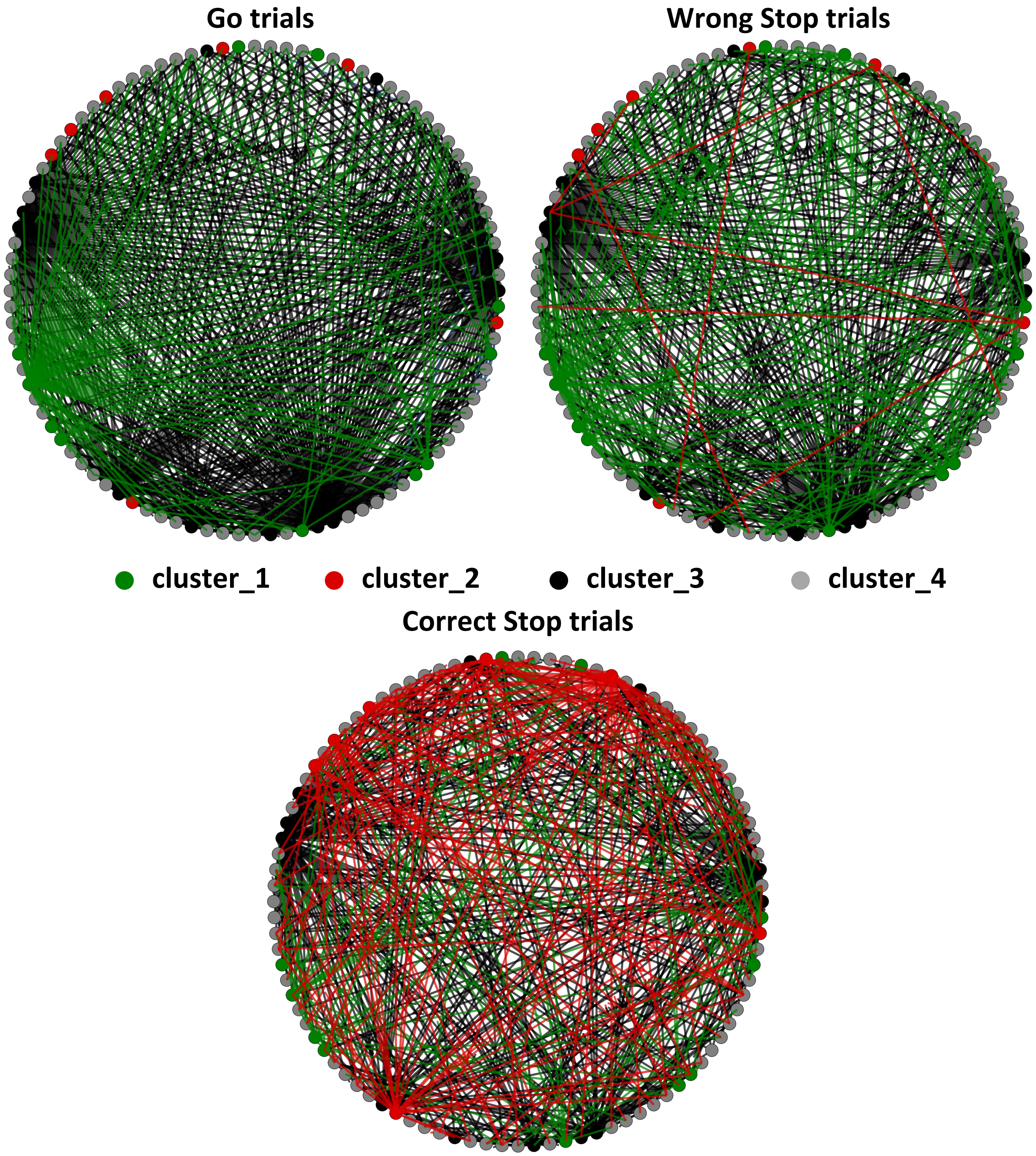

Figure S4. Detail of the PMd information network of Go, wrong and correct Stop trials for data in Fig.4. Each node and the respective connections are colour coded accordingly to the corresponding cluster (see legend). The coordinates of each node on the circle are the same as Fig. 4

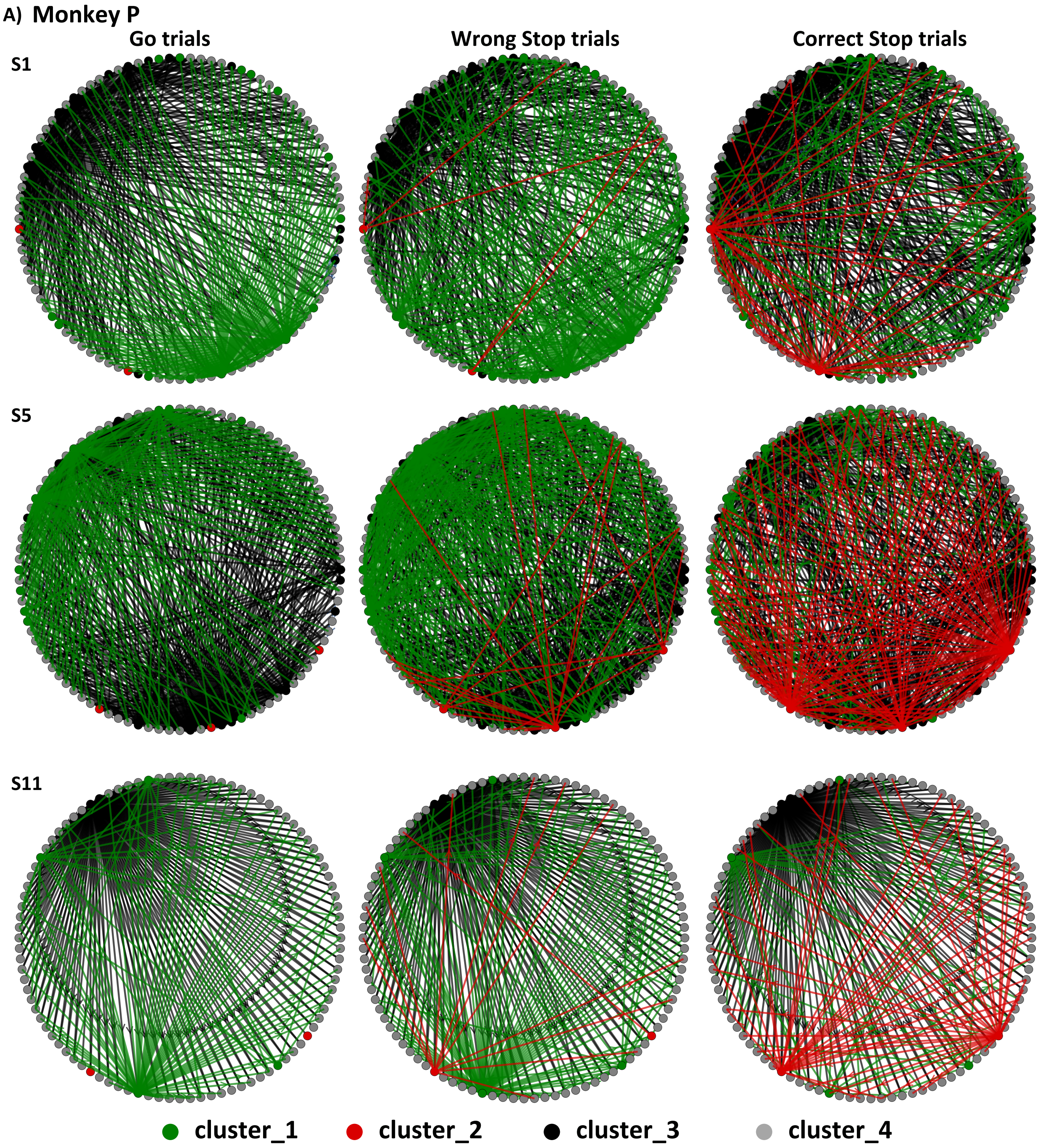
A) Monkey P
Go trials
Wrong Stop trials
Correct Stop trials
S1
S5
S11
cluster_1
cluster_2
cluster_3
cluster_4

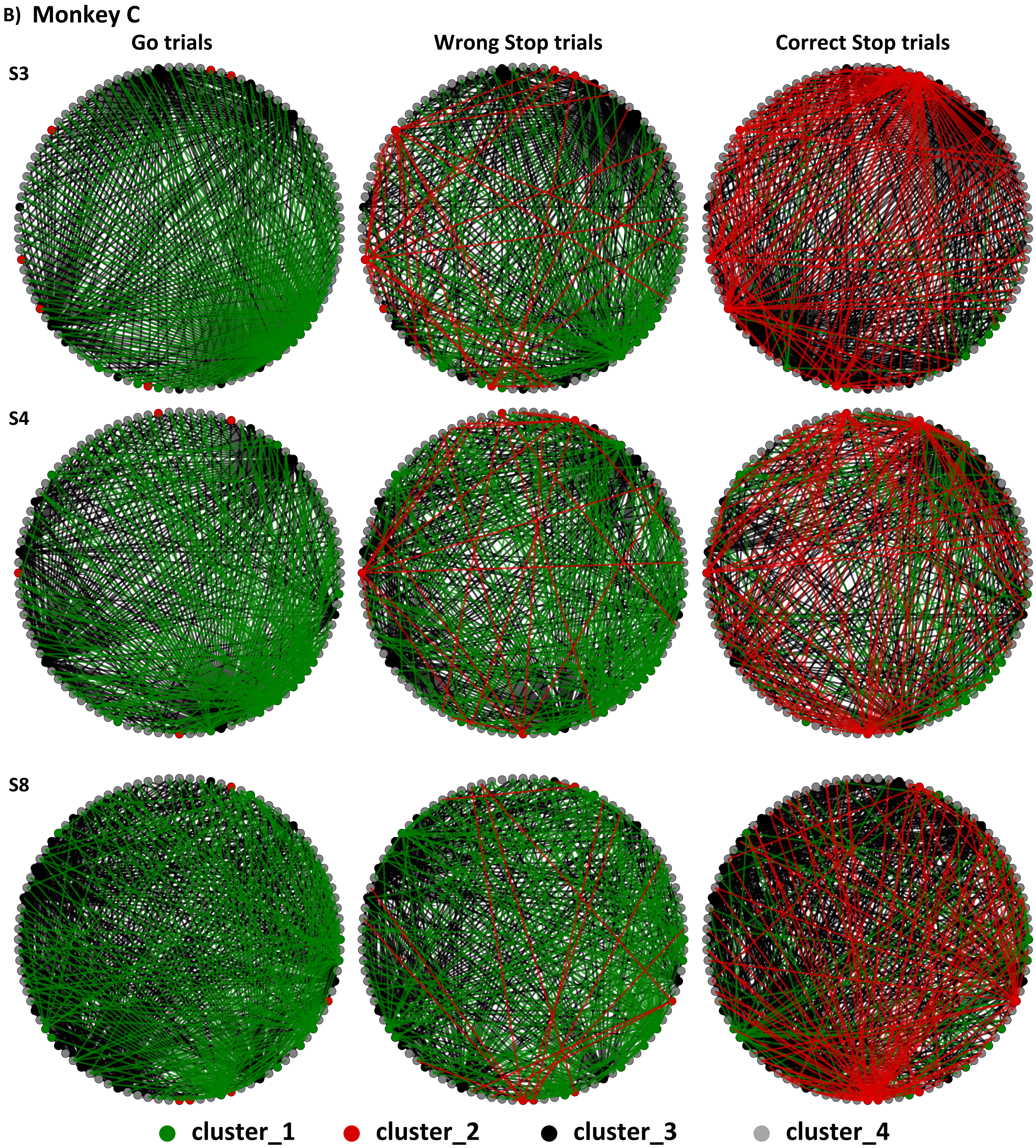

Figure S5. Detail of the PMd information network of Go, wrong and correct Stop trials for three example sessions for Monkey P (panel A) and Monkey C (panel B). Each node and the respective connections are colour coded accordingly to the corresponding cluster (see legend). The coordinates of each node on the circle are preserved for each network. As in Fig. 4 and Fig. S4, only the 20% of strongest connections are shown for illustrative purposes. The example sessions shown here are those that, given the network's link density, were best suited to be presented in this graphical representation. The coordinates of each node on the circle are the same as Fig. 4 and Fig. S4 and are preserved across behavioral conditions and animals.

Behavioural Results							
**Monkey P**							
S	$\overline{RT}_{Go}$	$\overline{RT}_{Wr}$	$\overline{SSD}_{CS}$	$\overline{SSD}_{Wr}$	$SSRT$	$P_{inhibit}$	*p-value*
1	590 ms	559 ms	273 ms	337 ms	285 ms	0.52	$p < 0.05$
2	584 ms	565 ms	305 ms	408 ms	227 ms	0.50	$p < 0.05$
3	618 ms	592 ms	335 ms	430 ms	238 ms	0.52	$p < 0.05$
4	573 ms	549 ms	301 ms	401 ms	221 ms	0.50	$p < 0.01$
5	572 ms	540 ms	293 ms	379 ms	236 ms	0.50	$p < 0.05$
6	643 ms	625 ms	402 ms	496 ms	195 ms	0.51	$p < 0.05$
7	600 ms	568 ms	340 ms	441 ms	209 ms	0.49	$p < 0.01$
8	664 ms	641 ms	469 ms	525 ms	170 ms	0.57	$p < 0.01$
9	786 ms	753 ms	528 ms	627 ms	213 ms	0.55	$p < 0.01$
10	672 ms	619 ms	421 ms	500 ms	216 ms	0.57	$p < 0.01$
11	766 ms	721 ms	497 ms	504 ms	230 ms	0.51	$p < 0.01$
12	868 ms	853 ms	675 ms	746 ms	160 ms	0.55	$p < 0.05$
**Monkey C**							
1	598 ms	523 ms	322 ms	383 ms	250 ms	0.57	$p < 0.01$
2	539 ms	460 ms	382 ms	378 ms	158 ms	0.60	$p < 0.05$
3	561 ms	522 ms	318 ms	403 ms	207 ms	0.58	$p < 0.05$
4	672 ms	631 ms	423 ms	507 ms	217 ms	0.60	$p < 0.05$
5	638 ms	612 ms	401 ms	512 ms	189 ms	0.56	$p < 0.05$
6	579 ms	533 ms	308 ms	426 ms	224 ms	0.60	$p < 0.01$
7	667 ms	620 ms	383 ms	498 ms	240 ms	0.59	$p < 0.05$
8	697 ms	672 ms	430 ms	546 ms	223 ms	0.60	$p < 0.05$
9	688 ms	662 ms	401 ms	547 ms	230 ms	0.60	$p < 0.05$

Table S1. Behavioural results. S, index of the recording session. $\overline{RT}_{Go}$, mean reaction time of Go trials. $\overline{RT}_{Wr}$, mean reaction time of wrong Stop trials. $\overline{SSD}_{CS}$, mean SSD of correct Stop trials. $\overline{SSD}_{Wr}$, mean SSD of Wrong Stop trials. $SSRT$, Stop signal reaction time. $P_{inhibit}$, inhibition probability. The *p-values* result from the independence; Kolmogorov-Smirnov test, $p < 0.05$ between RT_{Go} and RT_{Wr} distributions. (**Go**: Go trials; **WS**: wrong Stop trials; **CS**: correct Stop trials)

I matrix details				
**Monkey P**				
**Go trials**	cluster_1	cluster_2	cluster_3	cluster_4
cluster_1	/	0.08 ± 0.03	0.12 ± 0.02	1.8 ± 0.2
cluster_2	0.04 ± 0.005	/	0.04 ± 0.01	0.7 ± 0.06
cluster_3	0.24 ± 0.04	0.14 ± 0.04	/	4.1 ± 0.9
cluster_4	0.01 ± 0.005	0.01 ± 0.003	0.02 ± 0.007	/
**Wrong Stop trials**	cluster_1	cluster_2	cluster_3	cluster_4
cluster_1	/	0.05 ± 0.008	0.1 ± 0.01	1.6 ± 0.3
cluster_2	0.05 ± 0.008	/	0.045 ± 0.01	0.7 ± 0.1
cluster_3	0.25 ± 0.04	0.13 ± 0.04	/	4.0 ± 1.2
cluster_4	0.02 ± 0.004	0.008 ± 0.001	0.02 ± 0.005	/
**Correct Stop trials**	cluster_1	cluster_2	cluster_3	cluster_4
cluster_1	/	0.01 ± 0.003	0.05 ± 0.009	0.75 ± 0.01
cluster_2	0.2 ± 0.003	/	0.12 ± 0.02	1.9 ± 0.2
cluster_3	0.28 ± 0.03	0.10 ± 0.02	/	3.5 ± 0.9
cluster_4	0.02 ± 0.005	0.004 ± 0.001	0.02 ± 0.005	/
**Monkey C**				
**Go trials**	cluster_1	cluster_2	cluster_3	cluster_4
cluster_1	/	0.1 ± 0.012	0.11 ± 0.02	2.2 ± 0.45
cluster_2	0.016 ± 0.004	/	0.03 ± 0.005	0.53 ± 0.06
cluster_3	0.25 ± 0.06	0.18 ± 0.02	/	4.2 ± 0.9
cluster_4	$0.004 \pm 10^{-}4$	0.009 ± 0.001	0.008 ± 0.002	/
**Wrong Stop trials**	cluster_1	cluster_2	cluster_3	cluster_4
cluster_1	/	0.065 ± 0.013	0.13 ± 0.013	2.6 ± 0.24
cluster_2	0.02 ± 0.008	/	0.03 ± 0.005	0.66 ± 0.1
cluster_3	0.21 ± 0.05	0.18 ± 0.02	/	4 ± 0.7
cluster_4	0.014 ± 0.002	0.01 ± 0.002	0.009 ± 0.001	/
**Correct Stop trials**	cluster_1	cluster_2	cluster_3	cluster_4
cluster_1	/	0.02 ± 0.004	0.03 ± 0.008	0.72 ± 0.1
cluster_2	0.13 ± 0.015	/	0.10 ± 0.01	1.9 ± 0.2
cluster_3	0.3 ± 0.03	0.11 ± 0.02	/	3.8 ± 0.5
cluster_4	0.018 ± 0.002	0.007 ± 0.001	0.01 ± 0.001	/

Table S2. *I* matrix details. Values of I for each cluster and each behavioral condition averaged (mean ± SEM) over recording sessions. The symbol / marks the absence of self loops which are excluded from the analysis (see main text).

.

**I matrix *p-values***			
**Monkey P**			
$\boldsymbol{I_{i \to j}}$	$\boldsymbol{GO-CS}$	$\boldsymbol{GO-WS}$	$\boldsymbol{CS-WS}$
$C_1 \to C_2$	$p < 0.01$	$p = 1$	$p < 0.05$
$C_1 \to C_3$	$p < 0.01$	$p = 1$	$p < 0.01$
$C_1 \to C_4$	$p < 0.01$	$p = 1$	$p < 0.01$
$C_2 \to C_1$	$p < 0.01$	$p = 1$	$p < 0.05$
$C_2 \to C_3$	$p < 0.01$	$p = 1$	$p < 0.05$
$C_2 \to C_4$	$p < 0.01$	$p = 1$	$p < 0.01$
$C_3 \to C_1$	$p = 0.7$	$p = 1$	$p = 0.7$
$C_3 \to C_2$	$p = 0.5$	$p = 1$	$p = 0.8$
$C_3 \to C_4$	$p = 1$	$p = 1$	$p = 1$
$C_4 \to C_1$	$p = 0.2$	$p = 1$	$p = 0.6$
$C_4 \to C_2$	$p = 0.3$	$p = 1$	$p = 0.6$
$C_4 \to C_3$	$p = 1$	$p = 1$	$p = 15$
**Monkey C**			
$C_1 \to C_2$	$p < 0.01$	$p = 0.7$	$p < 0.05$
$C_1 \to C_3$	$p < 0.01$	$p = 0.9$	$p < 0.05$
$C_1 \to C_4$	$p < 0.01$	$p = 0.7$	$p < 0.01$
$C_2 \to C_1$	$p < 0.01$	$p = 1$	$p < 0.01$
$C_2 \to C_3$	$p < 0.05$	$p = 1$	$p < 0.05$
$C_2 \to C_4$	$p < 0.01$	$p = 1$	$p < 0.01$
$C_3 \to C_1$	$p = 0.6$	$p = 1$	$p = 0.3$
$C_3 \to C_2$	$p = 0.1$	$p = 1$	$p = 0.1$
$C_3 \to C_4$	$p = 1$	$p = 1$	$p = 1$
$C_4 \to C_1$	$p = 0.2$	$p = 0.3$	$p = 0.2$
$C_4 \to C_2$	$p = 0.8$	$p = 1$	$p = 0.3$
$C_4 \to C_3$	$p = 1$	$p = 1$	$p = 15$

Table S3. *p-values* for the interactions reported in Table S2. $I_{i \to j}$: interaction term between cluster i and cluster j. *p-values* are obtained from Kruskal-Wallis test over recording sessions, Bonferroni corrected for multiple comparisons. ($\boldsymbol{C_1}$: cluster_1; $\boldsymbol{C_2}$: cluster_2; $\boldsymbol{C_3}$: cluster_3; $\boldsymbol{C_4}$: cluster_4; **Go**: Go trials; **WS**: wrong Stop trials; **CS**: correct Stop trials).

ANOVA Results			
**Monkey P**			
S	N_{Go-CS}	N_{Go-WS}	N_{CS-WS}
1	70%	0	51%
2	77%	0.01%	66%
3	65%	0	42%
4	69%	0	60%
5	62%	0.01%	45%
6	78%	0	65%
7	79%	0.01%	76%
8	92%	0	84%
9	58%	0	43%
10	68%	0	44%
11	68%	0	63%
12	70%	0	68%
**Monkey C**			
1	98%	0	98%
2	92%	0%	92%
3	66%	0	67%
4	67%	0	67%
5	94%	0%	93%
6	92%	0	92%
7	76%	0%	76%
8	85%	0	84%
9	89%	0	88%

Table S4. ANOVA results. S, index of the recording session. N_{Go-CS}, number of modules with $p < 0.01$ between Go and correct Stop trials. N_{Go-WS}, number of modules with $p < 0.01$ between Go and wrong Stop trials. N_{CS-WS}, number of modules with with $p < 0.01$ between correct and wrong Stop trials. All Ns are in percentage over the total number of moduels. *p-values* are Bonferroni corrected for multiple comparisons. (**Go**: Go trials; **WS**: wrong Stop trials; **CS**: correct Stop trials)

Clusters Composition	
**Monkey P** $N = 96$	
**Cluster**	$\mu \pm SEM$
cluster_1	9.3 ± 1.06
cluster_2	3.6 ± 1.4
cluster_3	11.8 ± 1.6
cluster_4	71.3 ± 2.3
**Monkey C** $N = 96$	
**Cluster**	$\mu \pm SEM$
cluster_1	7.4 ± 0.8
cluster_2	4.1 ± 0.6
cluster_3	9.4 ± 1.1
cluster_4	75.0 ± 1.8

Table S5. Clusters composition. For each monkey the composition of clusters averaged (mean ± SEM) over recording sessions is reported. N is the number of modules available.

VD_{out}			
**Monkey P** $N = 12$			
**Cluster**	**Go trials**	**Wrong Stop trials**	**Correct Stop trials**
cluster_1	87.74 ± 0.77	52 ± 1.75	32.86 ± 1.33
cluster_2	48.74 ± 3.5	43.23 ± 3.11	67.14 ± 2.43
cluster_3	92.02 ± 0.46	68.6 ± 1.54	72.77 ± 1.34
cluster_4	26.04 ± 0.81	14.31 ± 0.40	13.96 ± 0.39
**Monkey C** $N = 9$			
**Cluster**	**Go trials**	**Wrong Stop trials**	**Correct Stop trials**
cluster_1	77.73 ± 1.49	35.56 ± 1.80	24.44 ± 1.33
cluster_2	37.78 ± 2.09	17.27 ± 1.32	52.08 ± 1.84
cluster_3	84.82 ± 1.27	54.95 ± 2.50	69.62 ± 1.92
cluster_4	17.06 ± 0.59	8.37 ± 0.24	10.28 ± 0.31

Table S6 Details of VD_{out} across behavioral conditions. For each monkey VD_{out} values averaged (mean ± SEM) over recording sessions are reported. The cluster_3 showed the highest values of VD_{out} compared to other clusters in all behavioural conditions. The cluster_1 and the cluster_2 showed the second highest VD_{out} values during both Go and wrong Stop trials and correct Stop trials respectively; Kruskal-Wallis, $p < 0.01$ Bonferroni corrected for multiple comparisons. **N**: number of session available.
